# Rat thyroid graft transplantation after cryopreservation with scintigraphic standardization for an experimental study

**DOI:** 10.1016/j.clinsp.2022.100065

**Published:** 2022-06-26

**Authors:** Alberto Schanaider, Thiago Barboza, Marcel Vasconcellos, Gabriel Gutfilen-Schlesinger, Sergio Augusto Lopes de Souza

**Affiliations:** a Programa de Pós-Graduação em Ciências Cirúrgicas, Departamento de Cirurgia, Faculdade de Medicina, Universidade Federal do Rio de Janeiro, Rio de Janeiro, RJ, Brazil; b Departamento de Radiologia, Universidade Federal do Rio de Janeiro, Faculdade de Medicina, Rio de Janeiro, RJ, Brazil

**Keywords:** Scintigraphy, Thyroidectomy, Single photon emission computed tomography, ^99m^Tc, Nuclear Medicine, CG, Control group, HTG, Hypothyroidism group, TG, Transplanted group, T4, l-thyroxine, 99mTcO4, Pertechnetate, NM, Nuclear Medicine

## Abstract

**Purpose:**

Budget cuts among other factors undermine the use of state-of-the-art equipment by many research groups. This doesn't mean that their scientific data are not reliable or top-notch. Resort to adaptations is a recurrent need in their reality. The aim of this study was to assess whether scintigraphy with ^99m^TcO_4_ is effective in evaluating the functionality of thyroid grafts after cryopreservation in rats.

**Material and methods:**

24 rats were randomly distributed into 3 groups: Control Group (CG), without surgical procedure, Hypothyroidism Group (HTG), submitted to total thyroidectomy, and Transplanted Group (TG), with total thyroidectomy and cryopreservation of the thyroid gland for 7 days followed by grafting of a thyroid lobe. A protocol using a gamma camera imaging was conducted fourteen weeks after transplantation, and the whole body ^99m^Tc, focusing on the topic of heterotopic thyroid uptake was evaluated.

**Results:**

The images acquired had good quality with no noise and artifacts that could jeopardize its analysis. On the 14th day, HTG displayed no thyroid uptake, and the TG had a clear uptake of the thyroid graft in the topography of the biceps femoris muscle. Presented data also showed that both equipment spatial resolution and alignment (4.375 mm) did not interfere with the physiological uptake of ^99m^Tc by the thyroid graft.

**Conclusion:**

The viability and functionality of cryopreserved thyroid autotransplantation in rats who underwent total thyroidectomy were successfully accessed by the scintigraphy protocol developed.

## Highlights

• Adaptations in validated methods are a recurrent trend due to limited budgets which does not diminish their functionality.
• Scintigraphy with ^99m^TcO_4_ is effective in evaluating the functionality of thyroid grafts after cryopreservation in rats.
• It is possible to adapt human SPECT for other animals for clinical and research purposes.

## Introduction

Antithyroid medication, surgery, and radioiodine ablation are commonly prescribed for thyroid diseases like Graves’ disease, thyroiditis, goiter, and other causes of hyperthyroidism. It is not uncommon that these therapeutic interventions could lead to hypothyroidism, triggering metabolic syndromes, cardiac dysfunction, and anemia among other symptoms that require a permanent hormonal l-Thyroxine (T4) replacement.^1^, ^2^, ^3^, ^4^

Halsted, in 1909, suggested that an autologous transplant of the parathyroid glands could suppress the hormonal therapy in those patients.^1^ This procedure was later validated in the scientific literature.^2^, ^3^, ^4^ More recently, cryopreservation and thyroid autograft were improved and such surgical alternatives may be useful for a patient's treatment.^5^, ^6^, ^7^, ^8^ Functional imaging studies of the transplanted organ are scarcely found in the respective literature.

Nuclear Medicine (NM) approaches are minimally invasive and use radionuclides for physiological analysis. Basically, instilled radioactive material has its emissions measured for organ functionality evaluation.^9^,^10^ Radionuclide choosing will depend on the purpose of the study (diagnostic or therapeutic), costs, and radionuclide/scanner availability. For thyroid imaging, Iodine (I) −123 and −131 and Pertechnetate(^99m^TcO_4_) can be used in Single-Photon Emission Computed Tomography (SPECT) scan.^9^,^11^,^12^ I-123 shows better features although it's got a more costly production than ^99m^TcO_4_.^13^, ^14^, ^15^, ^16^ Besides being cost-effective, ^99m^TcO_4_ is more suitable than I-123, because of a smaller time gap for imaging acquisition, respectively 15‒30 min instead of 4‒24 h after injection.^12^,^15^,^17^ Both radionuclides share the same embodiment mechanism through the sodium/iodide symporter. Hence, its accumulation in the cell is a useful function assessment agent.^18^, ^19^, ^20^, ^21^

In the present study, the authors investigated if human scintigraphy with ^99m^TcO_4_ could be standardized and adapted to evaluate effectively thyroid grafts after cryopreservation, in rats.

## Material and methods

### Study design

This study follows the ARRIVE Guidelines 2.0 for reporting animal research. All the procedures in this study were approved by the Ethics Committee on the Use of Animals (CEUA) of the Federal University of Rio de Janeiro (UFRJ), in accordance with the guidelines of the International Care and Use Committee of the National Institutes of Health, and Guide for the Care and Use of Laboratory Animals (National Research Council ‒ USA, 2011) and complying with Brazilian national regulations for animal experiments.

It was used in 24 adult Wistar rats (*Rattus norvegicus albinus*, Rodentia Mammalia ‒ Berkenhaout, 1769), males, weighting 300±20 g, from the Center of Experimental Surgery of the Department of Surgery, School of Medicine, Federal University of Rio de Janeiro/UFRJ.

Animals were randomly distributed into 3 groups of 8 animals each. Control Group (CG), without surgical procedure, Hypothyroidism Group (HTG), submitted to total thyroidectomy, and Transplanted Group (TG), with total thyroidectomy and cryopreservation of the thyroid gland for 7 days followed by grafting of a thyroid lobe.

Sample size calculation was performed with G*Power® (Heinrich-Heine-Universität, DE), based on a prior pilot study with 8 individuals in each of the four groups (*n* = 32). The quantitative variable compared between the groups was free T4 (ng/nL), effect size to be 4.84 (SD ± 0.6 ng/dL) statistical significance of 0.05.

### Operative technique for total thyroidectomy and graft transplantation

The anesthetic procedure consisted of injecting an intraperitoneal solution of 10% ketamine hydrochloride (100 mg/kg) with 2% xylazine hydrochloride (10 mg/kg). After asepsis and antisepsis care, a 1.18-inch longitudinal skin incision was made in the ventral cervical region using a surgical microscope, with 10 ×  magnification, in order to perform a total thyroidectomy. The left lobe was implanted in the biceps femoris muscle of the right hind leg (of the TG). Muscle and skin layers were closed in simple interrupted stitches, respectively with 4‒0 polyglycolic acid and 3‒0 monofilament nylon threads.

### Cryopreservation of the thyroid gland

After removal, the thyroid gland was kept in a 1 × sterile PBS solution (pH 7.4). In a laminar flow chamber, a nutrient solution containing 43% RPMI 1640 (Cultilab, Campinas, SP, BR), 50% bovine fetal serum (Sigma, St Louis, Mo, USA), and 7% DMSO (dimethylsulfoxide) was packed in a cryotube previously cooled in ice, as mentioned by Taylor and cols. (2019).^27^ The cryotube was then placed in a freezer (−4 °C) for 1 hour. At the end of the process, the sample was identified and stored for seven days in liquid nitrogen at −196 °C. To thaw the samples the group used a 37 °C water bath until the sample reached room temperature and two washes were performed with sterile PBS.

### Scintigraphy

Scintigraphy imaging was conducted with a dual-head detector GE Millennium MG, H30002L model from the Departamento de Radiologia, Hospital Universitário Clementino Fraga Filho da Universidade Federal do Rio de Janeiro (HUCFF/UFRJ). Fourteen weeks after transplantation, ^99m^Tc whole body uptake performed with the aforementioned gamma camera was analyzed.

All rats were intraperitoneally anesthetized prior to intravenous (iv) catheter 24 G (Angiocath™, BD) insertion in the lateral tail vein for radioisotope (12.95 MBq) injection. Syringe radioactivity was measured after injection, for actual dose quantification. Ten minutes after the ^99m^Tc administration, furosemide (2 mg/Kg) was injected. Afterward, the animals were positioned in dorsal decubitus for planar imaging acquisition.

Small animal's planar imaging acquisition requires (i) Low Energy High Resolution (LEHR) collimator; (ii) duration of the procedure of 10 min. (iii) standardized parameters: matrix 512 × 512, visual field augmentation: 2.00; detectors were positioned at a 105 mm distance and table at 764 mm vertical distance.

### ^99m^TcO_4_ ex vivo biodistribution

By the 14th week, the animals were weighted (Shimadzu AY220®, 210 g/0,0001 g, Kyoto, JP) and euthanized (300 mg/kg ketamine and 30 mg/kg xylazine) for *ex vivo* organ radioactivity analysis (heart, lungs, intestines, kidney, stomach, pancreas, spleen, trachea and thyroid), left biceps femoris in the CG; graft and contralateral femoral biceps in the TG and lastly, blood was drawn from both groups.

Dissected organs were also weighed and had their radioactivity analyzed through a gamma-counter (2470 WIZARD^2^ (TM) ‒ PerkinElmer Inc.®, Waltham, MA, EUA). Percentual radioactivity was calculated by dividing emission per gram of extracted organ/tissue.

### Statistical analysis

The Shapiro-Wilk normality test, the Analysis of Variance (ANOVA), followed by the Tukey test were used. In all tests, a 95% Confidence Interval (95% CI) and 5% statistical significance (*p* < 0.05) were established. The analyses were performed using the statistical program SPSS® version 22.0 (Belmont, CA, USA).

## Results

### Scintigraphic examinations

**Fig. 1:**
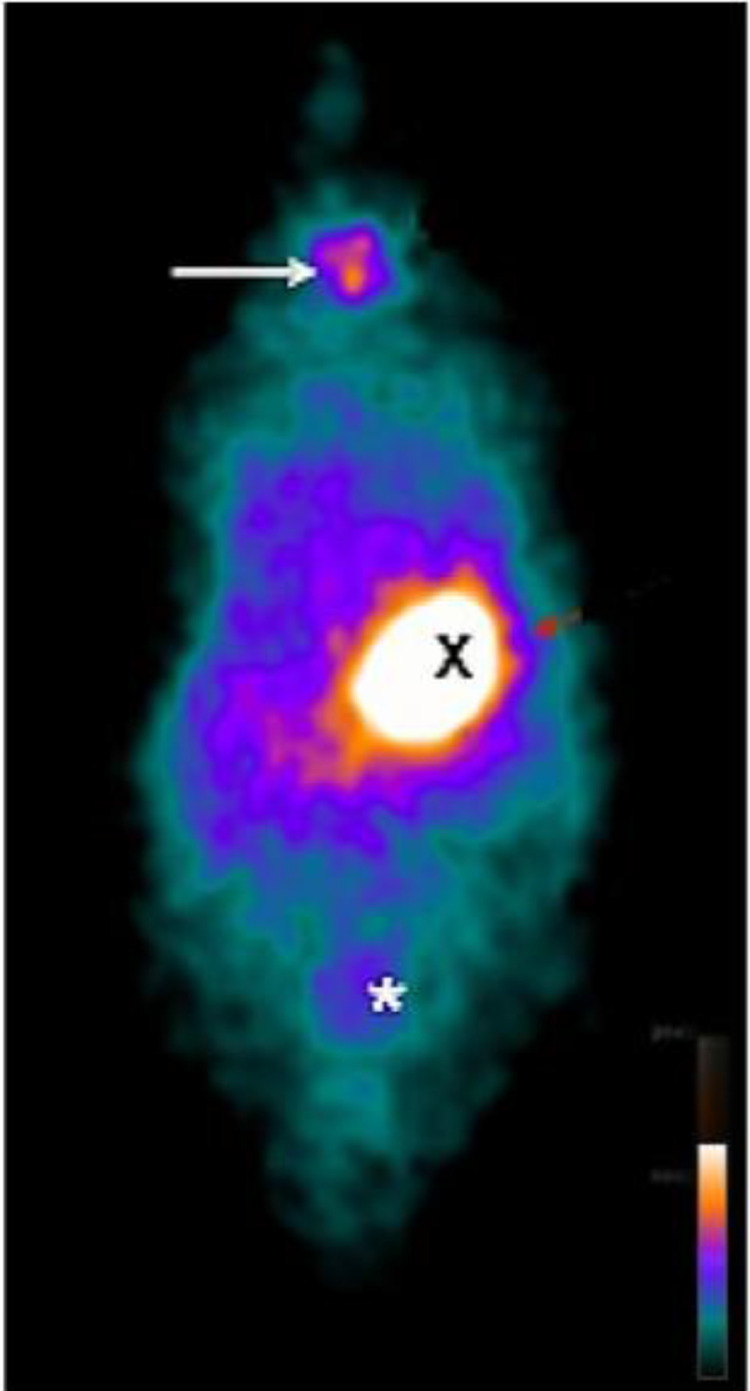
Whole body planar ^99m^Tc scintigraphy in CG of rats. ^99m^Tc biodistribution on the 14th day revealing higher uptake in thyroid (arrow), stomach (X) and bladder (*).

**Fig. 2:**
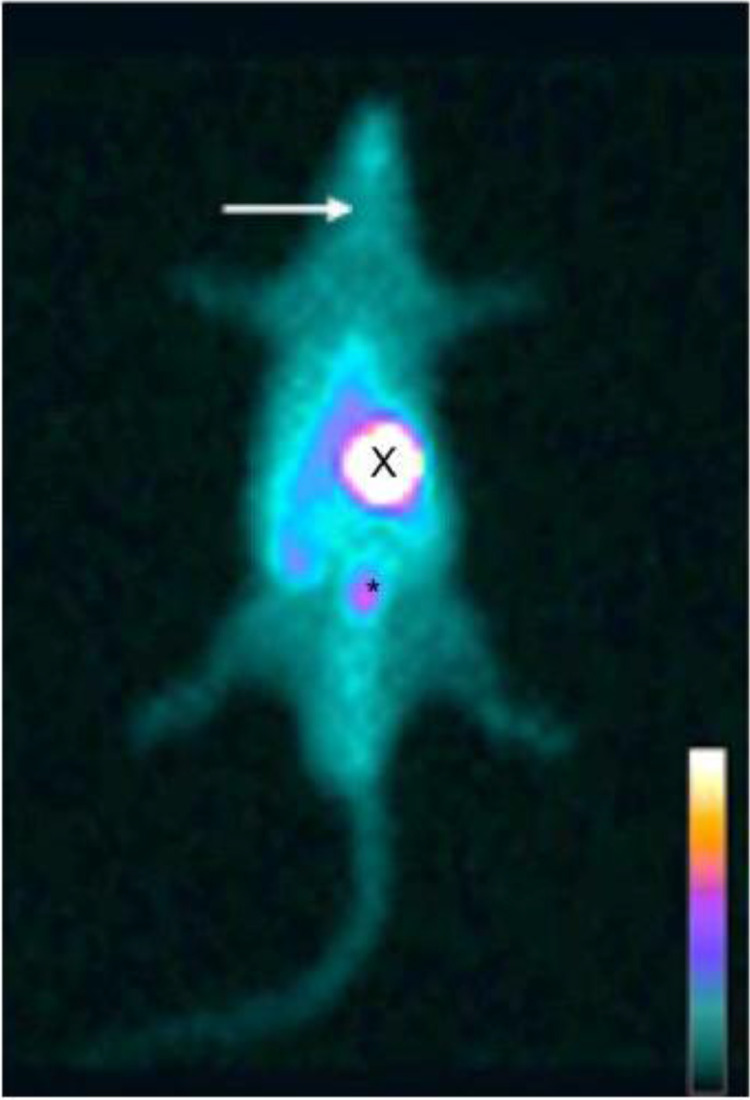
Whole body planar ^99m^Tc scintigraphy in thyroidectomized rats. ^99m^Tc biodistribution on the 14th day revealing no uptake on thyroid topography (arrow), while radioactive accumulation is seen on stomach (X), and bladder (*).

**Fig. 3:**
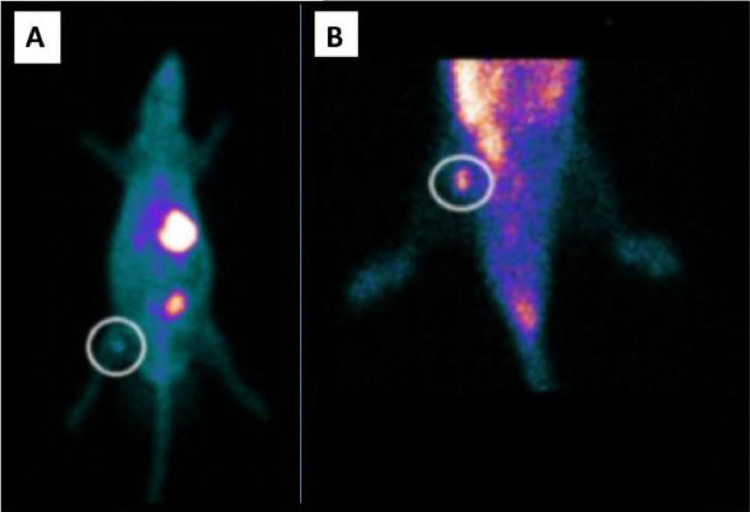
Whole body planar ^99m^Tc scintigraphy in TG rats. ^99m^Tc biodistribution on the 14th day revealing (A) heterotopic graft with a higher uptake on the right thigh (circle) and stomach (X), and bladder radioactive accumulation (*). (B) ROI centered on the right hind leg showing higher uptake (circle).

On the 14th week, in CG of rats, the thyroid, the stomach, and the bladder emissions had a higher uptake (Fig. 1). In HTG of animals, there was no thyroid uptake (Fig. 2). In TG, uptake of the thyroid graft in the topography of the biceps femoris muscle of the right hind leg (circle) was highlighted (Fig. 3).

**Fig. 4:**
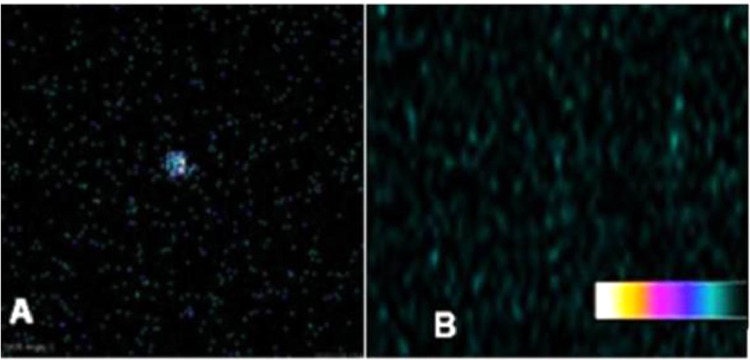
Ex vivo graft vs. biceps femoris muscle ^99m^Tc-biodistribution. 10-minute ^99m^Tc-planar scintigraphy biodistribution on the 14th day revealing (A) excinded thyroid graft with radionuclide accumulation. (B) Previously attached graft region, biceps femoris muscle, after graft excision with no radioactive emission.

In order to differentiate thyroid graft from muscle emissions, a ^99m^Tc scintigraphy study was performed comparing the *ex vivo* graft emission with the contralateral muscular portion in the same graft topography (Fig. 4).

### ^99m^Tc radioisotope biodistribution analysis

**Fig. 5:**
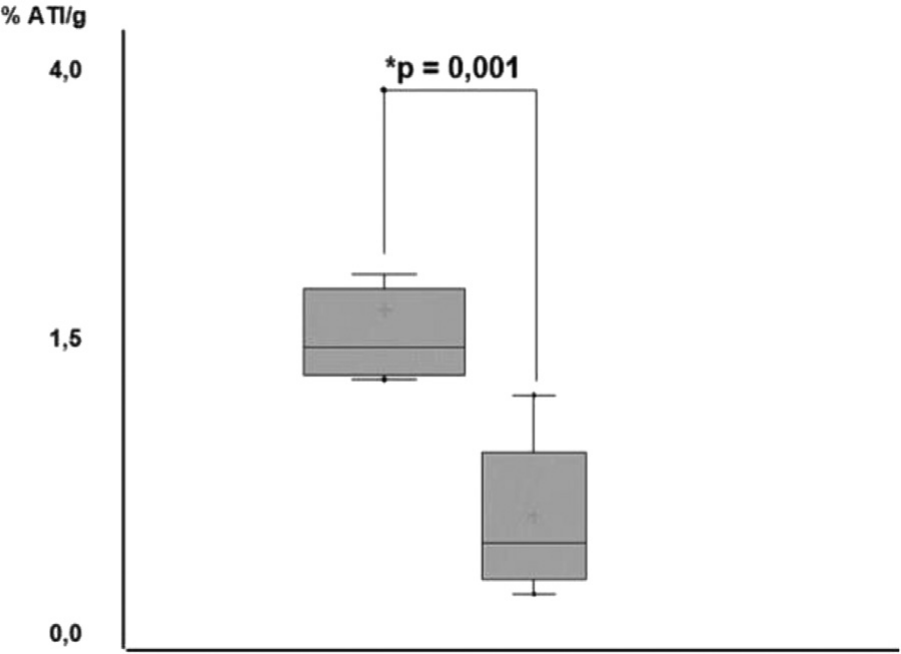
N-fold boxplot of ^99m^Tc uptake in TG graft vs. contralateral femoris biceps muscle. Graft uptake in TG was statistically higher (*p* = 0.001) than contralateral femoris biceps of the same group.

After scintigraphy images acquisition, the study performed individual organ and tissue emission analyses. Among the groups, biceps femoris grafts of the TG achieved a higher radionuclide accumulation (*p* < 0.001); (Table 1) when compared with those found in the contralateral muscle of the same animals (Fig. 5). After graft excision, the comparison of isotopic emissions on biceps femoris muscle between CG and TG had no statistical significance (*p* > 0.05).

**Table 1:** ^99m^Tc percentage uptake in thyroid (CG), Thyroid Graft (TG).

	CG	TG
Organs/Tissue	mean ± SD (%)	mean ± SD (%)
Thyroid	4.41 ± 0.51	‒
Thyroid graft	‒	1.43 ± 0.91
left biceps femoris (contralateral)	0.45 ± 0.16	0.60 ± 0.11^a^

CG, Control Group; TG, Transplantation Group; SD, Mean standard deviation; TG, Thyroid graft vs. left biceps femoris (contralateral) = ^a^*p* < 0.001.

No statistical difference (*p* > 0.05) on the other organs/tissues comparisons between CG and TG was found: blood (*p* = 0.11), heart (*p* = 0.27), right lung (*p* = 0.36), left lung (*p* = 0.26), liver (*p* = 0.63), spleen (*p* = 0.35), pancreas (*p* = 0.38), intestines (*p* = 0.61), right kidney (*p* = 0.28), left kidney (*p* = 0.06) e trachea (*p* = 0.17) (Table 2).

**Table 2:** Percentage ^99m^Tc uptake comparison on other organs/tissues between TG and CG.

	Control group	Transplanted group
Órgan/Tissue	mean ± SD	mean ± SD
Blood	2.16 ± 0.63	1.72 ± 0.64
Heart	1.79 ± 0.24	1.70 ± 0.28
Right lung	1.76 ± 0.50	1.66 ± 0.49
Left lung	1.76 ± 0.47	1.63 ± 0.34
Liver	1.79 ± 0.45	1.82 ± 0.32
Spleen	0.85 ± 0.29	0.80 ± 0.20
Pancreas	2.44 ± 0.76	2.55 ± 0.60
Stomach	13.51 ± 0.87	11.63 ± 1.09
Intestine	10.02 ± 1.66	9.74 ± 1.67
Right kidney	4.36 ± 0.29	4.50 ± 0.55
Left kidney	4.84 ± 0.59	4.33 ± 0.60

## Discussion

When the ^99m^Tc radioisotope enters the bloodstream, it is homogeneously distributed throughout the body, after which it is eliminated, mainly, by the digestive and renal systems, as attested in the scintigraphy exams previously performed.^19^,^22^,^23^

The present study involved preliminary protocols where the best radioisotope administration route (intraperitoneal or intravenous) and some temporal variables (5, 15, 30 and 60 min) in image acquisition were tested. The intravenous application of 0.35 µCi of ^99m^Tc, diluted in 0.5 mL of 0.9% sodium chloride solution, with isotopic scanning 10 min after administration, was considered the gold standard in obtaining high uptake images in the experimental model by acquiring better quality images with lower radiation doses.^35^

Directed small-animal SPECT, SPECT-CT/MRI novelties, or state-of-the-art technologies are mainly available in high-income countries or institutions with larger budgets and better economic development. This study was developed by adapting human SPECT equipment for animal research purposes, which is more available than small animal dedicated devices and encourages endeavors that consider older but still relevant techniques/equipment. The authors are aware of device limitations and not trying to compare dedicated technologies resolution and results with the one used in this study. Present data show that in spite of budget cuts and apparatus limitations, mods and proper adaptations can and must be done with trustworthy results.

It used a small animal planar scintigraphy with a LEHR collimator and followed a previously well-established 10-minute images acquisition protocol, 512 × 512 matrix and 2.0 zoom settings. The resulting images had good quality with no noise and artifacts that could jeopardize its analysis. Presented data demonstrated the equipment's spatial resolution and alignment (4.375 mm) did not interfere with the physiological uptake of the ^99m^Tc by the thyroid graft.

A full body scan of the HTG in the 14th week attested the absence of radioisotope uptake in the topography of the thyroid gland, which confirmed the efficacy of the surgical procedure. ^99m^Tc has both satisfactory sensitivity and high Positive Predictive Value (PPV), respectively 79% and 100%, for screening remaining tissue after total thyroidectomy.^23^ In addition, this radioisotope was chosen over the others, especially ^131^I, because it is not an organized substance (it does not remain inside the gland), interfering with hormonal values. In the 14th week, all animals of the TG presented uptake at the graft site (right thigh). This result exceeded those reported by Dobrinja^24^ in rats, who cited a successful rate of 70% in the 4th week after the graft had been implanted in the rectus abdominis muscle.

Based on the findings of Andreollo et al. (2012)^28^ 14-week period in a *R. norvegicus* life span correlates to 7-years time-lapse in the human life span. Also, studies^29^,^30^ have shown that in humans, this time period is sufficient to prove viability and function in an ectopic transplant (85% and 75%, respectively).

In week 14, the *ex vivo* analysis of the biodistribution of ^99m^Tc found significant differences in the percentage of radioactive activity per gram of tissues or organs when comparing the graft and the biceps femoral contralateral muscle between the animals of CG and TG (*p* = 0.001). This comparison was made in order to rule out a false positive uptake, attributed to the muscle tissue itself and which could generate an interpretation bias. Thus, the observed uptake biodistribution was a physiological small amount of radioisotope traces in the contralateral muscle tissue. In comparison to the data found at the graft site, it was significantly lower (*p* = 0.001) confirming the presence of functional thyroid tissue.

Roy^25^ described six patients with nontoxic multinodular goiter submitted to immediate autologous thyroid transplantation, who remained euthyroid at six months postoperatively, but only 45% became functional to radioisotopic uptake. Mohsen,^26^ after applying thyroid tissue emulsion to the thigh muscles at the same surgical time of a total thyroidectomy reported ^99m^Tc radioisotope uptake in all grafts. In fact, the present results endorse the presence of functional thyroid tissue.

Thyroid auto transplantation could be among other options a way to reduce postoperative hypothyroidism enhancing life quality for Graves’ disease patients, for instance. Although the autoimmune aspect of Graves’ disease plays a key role, it is found in the respective literature that patients with thyroid autotransplantation did not evolve with relapsing thyrotoxicosis.^25^^,^^29^, ^30^, ^31^, ^32^, ^33^

The American Thyroid Association 2015 guidelines among other studies support that another application of thyroid autotransplantation includes surgeries for lesser aggressive types or of low risk for the papillary differentiated thyroid carcinoma. Subtotal thyroidectomy or lobectomy with or without isthmectomy might be recommended for those cases.^33^,^34^

At last, ongoing research suggests that it might be a therapeutic option capable of suppressing permanently hormonal repositioning and its side effects on postoperative hypothyroidism.

Some limitations of the present study have to be considered. A clinical gamma camera was used. Thus, for the development of the experimental study, it was necessary to schedule the last available time in order to avoid contact with the hospital patients. In addition, the equipment must be properly cleaned after the animal procedure. However, it has been shown that human scintigraphy can be successfully adapted to evaluate thyroid cryopreserved grafts with ^99m^TcO_4_ in rats. It is also noteworthy that the standardized protocol developed in this study has other advantages considering that ^99m^TcO_4_ is not only more cost-effective than I-123, but also presents a significantly shorter time interval for imaging acquisition after injection.

## Conclusions

The viability and functionality of cryopreserved thyroid autotransplantation in rats were successfully accessed with a standardized ^99m^TcO_4_ protocol using human scintigraphy equipment adapted for experimental study.

## Ethics approval and consent to participate

All procedures using animals were approved by the institution's Animal Care and Use Committee (CEUA) and were conducted according to the International Care and Use Committee of the National Institutes of Health, Guide for the Care and Use of Laboratory Animals (National Research Council ‒ USA, 2011) and in agreement with Brazilian national regulations for animal experiments.

## Authors' contributions

All authors (AS, TB, MV, GGS, SALS) contributed equally to this project. All authors (AS, TB, MV, GGS, SALS) read and approved the final manuscript.

## Funding

This research was funded by Faperj grant CNE/E-26/202.921/2019.

## Data availability statement

The authors confirm that the data supporting the findings of this study are available within the article.

## Conflicts of interest

The authors declare no conflicts of interest.
